# PReS-FINAL-2253: A case series of HIV arthropathy in Cape Town

**DOI:** 10.1186/1546-0096-11-S2-P243

**Published:** 2013-12-05

**Authors:** K Webb, C Scott, N Brice

**Affiliations:** 1 Paediatric Rheumatology, University of Cape Town, Cape Town, South Africa

## Introduction

HIV arthropathy is well described in adults. Few studies have looked in depth at HIV arthropathy in children, and the characteristics of this entity have not been fully described.

## Objectives

To present a retrospective case series of children with HIV arthropathy in Cape Town.

## Methods

A retrospective chart review was undertaken for cases of HIV arthropathy at Red Cross Hospital, Tygerberg Hospital and Groote Schuur Hospital. Demographic, clinical, laboratory and treatment data were collected. WHO Staging for HIV was done.

## Results

15 patients were identified. 4 patients had insufficient data to be included.

10/11 Patients were boys.

Median age of presentation of arthritis was 10,2 yrs (2,8-13,4). Arthritis was the presenting feature of HIV in 8/11. WHO Stage 3 HIV was diagnosed in 9/11 patients. Polyarthritis (8/11) was the predominant rheumatological feature. None of the children had enthesitis. One child presented with dactylitis. Uveitis was present in 2/11. Three out of eleven had previously had TB and active TB was identified in 2 children. None of the children were on HAART at presentation. Median ESR was 122 (39-143) RF was done in 6/11 children and was negative in 6/6. HAART was initiated in 9/11 patients. Two patients were lost to follow up at our institution. All patients were treated with Ibuprofen. 9/11 were treated with chloroquine. Prednisone was used in 3/11 patients, methotrexate in 2/11 and sulphasalzine in 1/11. Intra-articular steroid injections were performed in 5/11 patients.

## Conclusion

In our case series, HIV arthropathy occurred in older boys, usually with late diagnosis of HIV, before HAART therapy and was the presenting feature of HIV in the majority. Polyarthritis was the most common mode of presentation. TB exposure was a frequent feature. Most children were treated with HAART therapy, ibuprofen, chloroquine.

## Disclosure of interest

None declared.

